# Temperature-Related Health Impacts: A Scoping Review and Benchmarking Exercise to Inform a Heat Action Plan

**DOI:** 10.5334/aogh.5016

**Published:** 2026-01-22

**Authors:** Caradee Y Wright, Muthise Bulani, Thandi Kapwata, Viwe Dikoko, Natasha Naidoo

**Affiliations:** 1 Climate Change and Health Research Programme, Environment and Health Research Unit, South African Medical Research Council, Pretoria, South Africa; 2 Department of Geography, Geoinformatics and Meteorology, University of Pretoria, Pretoria, South Africa; 3 Climate Change and Health Research Programme, Environment and Health Research Unit, South African Medical Research Council, Johannesburg, South Africa; 4 Department of Environmental Health, University of Johannesburg, Johannesburg, South Africa; 5 Climate Change and Health Research Programme, Environment and Health Research Unit, South African Medical Research Council, Cape Town, South Africa; 6 Climate Change and Health Research Programme, Environment and Health Research Unit, South African Medical Research Council, Durban, South Africa

**Keywords:** climate change, environmental health, heatwaves, hot days, vulnerability

## Abstract

*Background:* Global heating is associated with adverse health impacts necessitating the implementation of Heat Action Plans (HAPs) to protect communities. Gauteng in South Africa is the most populated province, housing three cities (i.e., Johannesburg, Ekurhuleni, and Pretoria) and 25% of the national population.

*Objective:* Given rising temperatures and projected increases in heatwaves and hot days, we gathered literature and case studies to inform the development of a Gauteng HAP.

*Methods:* We conducted a scoping review to inform baseline data on heat-related health impacts for Gauteng and South Africa too, followed by a benchmarking exercise that aimed to identify international best practices that may inform Gauteng’s plan. Benchmarking was done using Maharashtra (India), Victoria (Australia), and Khyber Pakhtunkhwa (Pakistan).

*Findings:* Thirty-six studies were included in the review, with 13 including Gauteng data and all showing impacts of heat on human health. Most studies applied epidemiological time series linking meteorological exposure (temperature/heat indices) and/or air pollutants (e.g., PM_2.5_, PM_10_, NO_2_, and O_3_) with health outcomes; applied remote-sensing, reanalysis, or station data for exposure assessment; and used regression or distributed lag models. The benchmarking exercise identified exemplars’ distinctive strengths: Victoria’s district thresholds keep activation simple and local—ideal for Gauteng’s heterogeneous microclimates across metros and townships. Maharashtra’s graded activation and clear departmental roles reduce ambiguity during multi-day heatwaves and thereby would help to align Gauteng Health, Infrastructure, Social Development departments. Khyber Pakhtunkhwa’s cooling-camp model shows practical, low-cost interventions of a low- and middle-income country that can be replicated at taxi ranks/clinics/malls during temperature peaks.

*Conclusions:* Insights from the literature and international exemplars provide a strong evidence base and adaptable models to guide a context-specific, multi-sectoral HAP for Gauteng that enhances preparedness, coordination, and community protection in a warming South Africa.

## Introduction

It has been projected that global land surface temperatures will exceed 1.5°C above preindustrial levels by 2027 [1, 2]. Accordingly, several reports from the Intergovernmental Panel on Climate Change (IPCC) have emphasized growing concerns that heat has affected vulnerable populations due to global warming [3, 4]. Heat is a significant global impact of climate change and under this scenario, children in Africa are set to be exposed to four to eight more heatwaves than people born in the 1960s [5].

South Africa, a semi-arid country [6] located within the mid-latitudes and subtropics, is characterized by pronounced climatic seasonality, with summer temperatures ranging from 15°C to 36°C [7]. Consequently, the country has already experienced several impacts of climate change, including pressing heat in the form of hot days and heatwaves [8]. South Africa is projected to experience a significant increase in hot days by 2100 [9]. Despite the centrality of rising heat in shaping agricultural practices, energy demand, health outcomes, and socio-economic planning, national heat-health adaptations remain largely unexplored [10].

The seasonality and heat indices vary greatly between the provinces in South Africa [11]. The complexity of the heat indices of these provinces is influenced by oceanic currents from the Indian and Atlantic Oceans [12], as well as heterogeneity in rainfall regimes [13], which complicates efforts to standardize heat-related classifications. These uncertainties underscore the need for province-specific heat-health adaptations to better characterize and manage the implications of heat in the diverse climatic zones in South Africa.

Gauteng’s climate has been described as a subtropical highland zone, a result of the province’s altitude on the Highveld plateau [14, 15]. This elevation moderates temperatures, resulting in warm to hot summers (e.g., average maximums of ~26°C in January) and cool, dry winters. Mean annual temperatures in Gauteng average approximately 19°C, peaking at about 28°C in December [16]. The “Humidex” (humidity index) is an index of well-being or “real feel” temperature calculated from the air temperature, relative humidity, and dew point temperature [16]. The humidex temperatures in Gauteng range from 18°C in July to 31°C in December [16].

Land surface temperatures are not only influenced by relative humidity and dew point temperature but also by the characteristics of the landscape, such as the presence or absence of vegetation and buildings. Gauteng is a province that has the two most affluent cities in South Africa, namely Johannesburg and Ekurhuleni. The influence of the urban heat island effect in these cities [17], as well as industrial and mining activities and the resultant poor air quality [18], is a mechanism influencing a rising temperature profile for Gauteng. Heat adaptation planning should be geared more prominently toward the most disproportionately affected people in these cities such as those who dwell in low-income areas [19, 20].

With the rising temperatures from global warming, lack of greenery in cities, and increasing levels of air pollution from industrial activities, there is an urgent need to understand fully the heat-related health implications of heat exposure in Gauteng, one of the most densely populated provinces in South Africa. This information will then be used to inform the development of a Heat Action Plan (HAP) for the province. We conducted a scoping review of the published literature on the heat-related health impacts in Gauteng province, as well as in South Africa. In addition, we carried out a benchmarking exercise with a select review of relevant international cases and methodologies for countries that have developed HAPs at a provincial level to inform the preparation of a Gauteng HAP for South Africa.

## Data and Methods

The scoping review followed the guidelines by the Joanna Briggs Institute guided by the Preferred Reporting Items for Systematic Reviews and Meta-Analyses Extension for Scoping Reviews (PRISMA-ScR) guidelines and the PRISMA-ScR checklist (Table S1) [21]. We searched four databases, namely, PubMed, ScienceDirect, Scopus, and Web of Science, for articles that met our inclusion criteria. Additional records were identified by screening the reference lists of included articles.

### Search strategy

The medical subject headings (MeSH) were used to develop the search strategy. The search terms used were (“heat” OR “hot days” OR “high temperatures”) AND (“health*” OR “well-being*”) AND Gauteng OR Johannesburg. The search was restricted to articles published in the English language up to and including 31 March 2025. The full search strategy is shown in the supplementary material (Table S2).

### Eligibility criteria

The population, exposure, context, outcome, and study design (PECOS) framework was followed to identify relevant articles (Table 1). The inclusion criteria consisted of articles that focused on humans of all ages, genders, and the geographical region of Gauteng or Johannesburg, including infants and pregnant women. Articles that measured at least one heat-related health outcome were included. Studies focusing on health risk assessment, exposure risk assessment, health impact assessment, and health modeling were excluded. Studies focusing on *in vivo*, animal, and environmental samples only were excluded. Occupational exposure was not considered. Reviews, reviews of review, conference abstracts, editorials, and dissertations were excluded.

**Table 1:** PECOS framing of the scoping review on heat and health in Gauteng.

**Population**	Studies conducted in Gauteng province (including Johannesburg)
**Exposure**	Studies that assessed environmental factors, agents, or exposures related to heat, heatwaves, and high temperature
**Concept/context**	Literature focused on heat in Gauteng province (including Johannesburg)
**Outcome**	Outcomes related to climate change, including climate change-related morbidities or mortalities
**Study design**	Primary studies/original research (qualitative and quantitative) that used or investigated the impact of heat on health outcomes

### Screening

The retrieved articles were uploaded to Endnote reference management software, and duplicates were removed. The articles were then exported to Rayyan, an online tool for the screening process. The remaining duplicates were removed. Three reviewers (NN, CYW, MB) independently screened the titles and abstracts of articles according to the inclusion and exclusion criteria (Table 2). This was followed by full-text screening of the relevant articles. Discrepancies were resolved through discussion until consensus was reached.

**Table 2:** Inclusion and exclusion criteria for the scoping review on heat and health in Gauteng.

INCLUSION	EXCLUSION
People of all ages	Animals
Weather-related elevated temperatures, including heatwaves and hot days	Non-weather-related heat such as body temperature, incubator temperature
Short-term and long-term exposure	No reference to heat exposure
Outdoor ambient temperature	Indoor temperature
Reference to air pollution/air quality that exacerbates heat	Air pollution/air quality but no reference to heat
Temperature, relative humidity, and other heat metrics	Exclusively studies with relative humidity or precipitation
Physical health: respiratory illnesses, cardiovascular issues, kidney conditions, and other illnesses; birth-related outcomes from pregnancy such as preterm birth, stillbirth, low birth weight, and birth defects	Studies on heat but no health-related outcomes

### Data extraction

A data extraction tool was developed and piloted against ten studies. Data were independently extracted from eligible studies by three reviewers (NN, CYW, MB). The data extracted from the studies included the following: first author, year of publication, study design, region, participant count, duration, methodology, main findings, limitations, policy recommendations, and future research.

### Quality assessment

A quality assessment was not performed since this is a scoping review, and the range in health outcomes and study design types is likely to be too large.

### Data synthesis and analysis

Descriptive tables were created to summarize the characteristics of the studies and synthesize the studies by the main themes, most likely by health outcome and by temperature ranges. The tables comprised: first author, year of publication, study design, region, participant count, duration, methodology, main findings, limitations, policy recommendations, and future research.

### Benchmarking exercise

**Figure 1:**
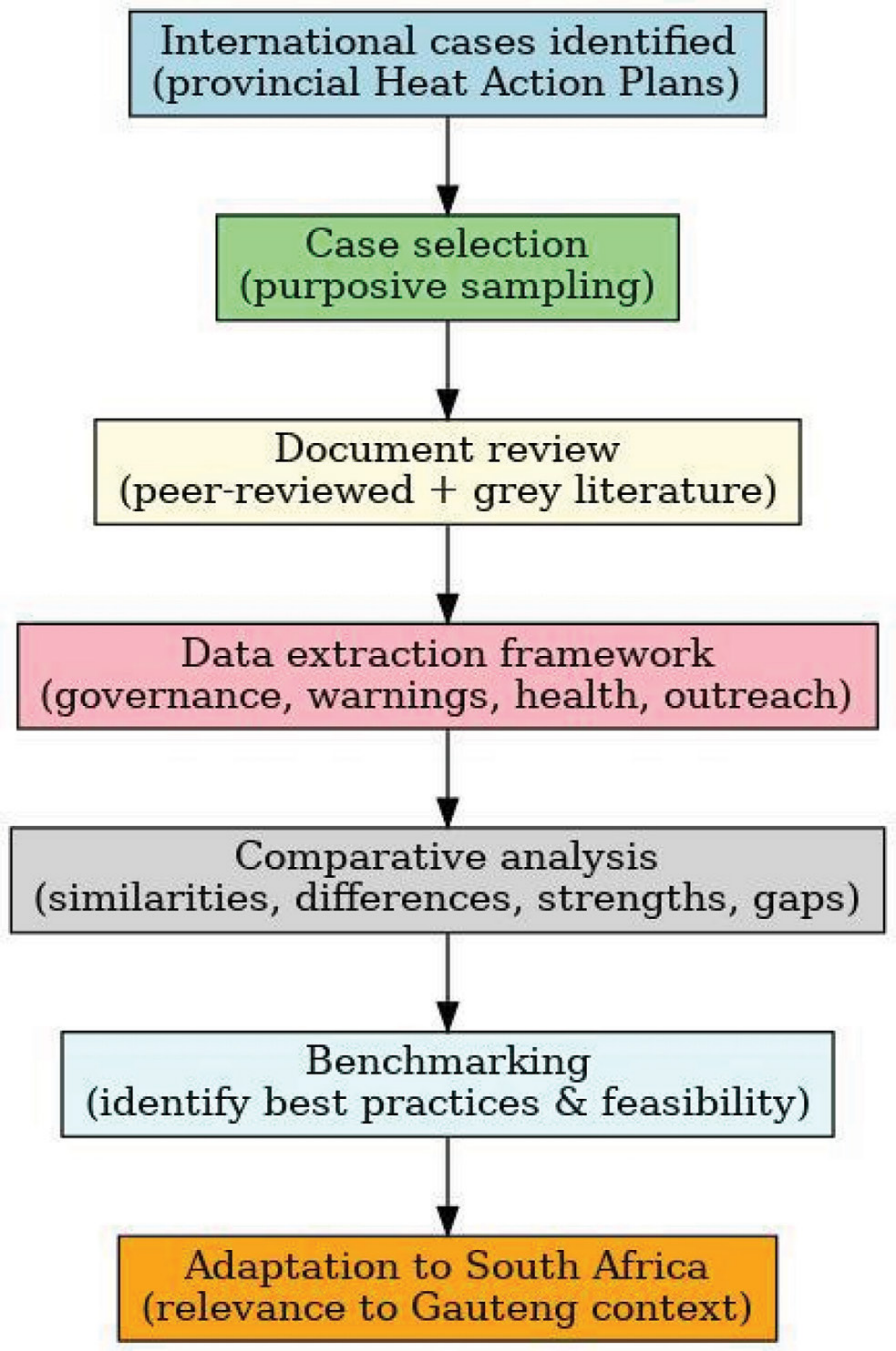
Methodological framework for the benchmarking exercise.

We conducted a benchmarking exercise through a selective review of international HAPs implemented at the provincial or sub-national level. Using a purposive sampling approach, we identified relevant cases from peer-reviewed and gray literature. A structured framework (Figure 1) guided data extraction on governance, early warning systems, health interventions, intersectoral collaboration, and community outreach. The findings were synthesized through comparative analysis to benchmark international best practices against the South African context, with the aim of informing the development of a Gauteng HAP. In addition to the selective review, we also considered countries that have developed HAPs at the national scale for six countries (i.e., France, England, the Netherlands, British Columbia, Japan, and Italy), and these findings are presented in the Supplementary Material.

## Results

### Study description

**Figure 2:**
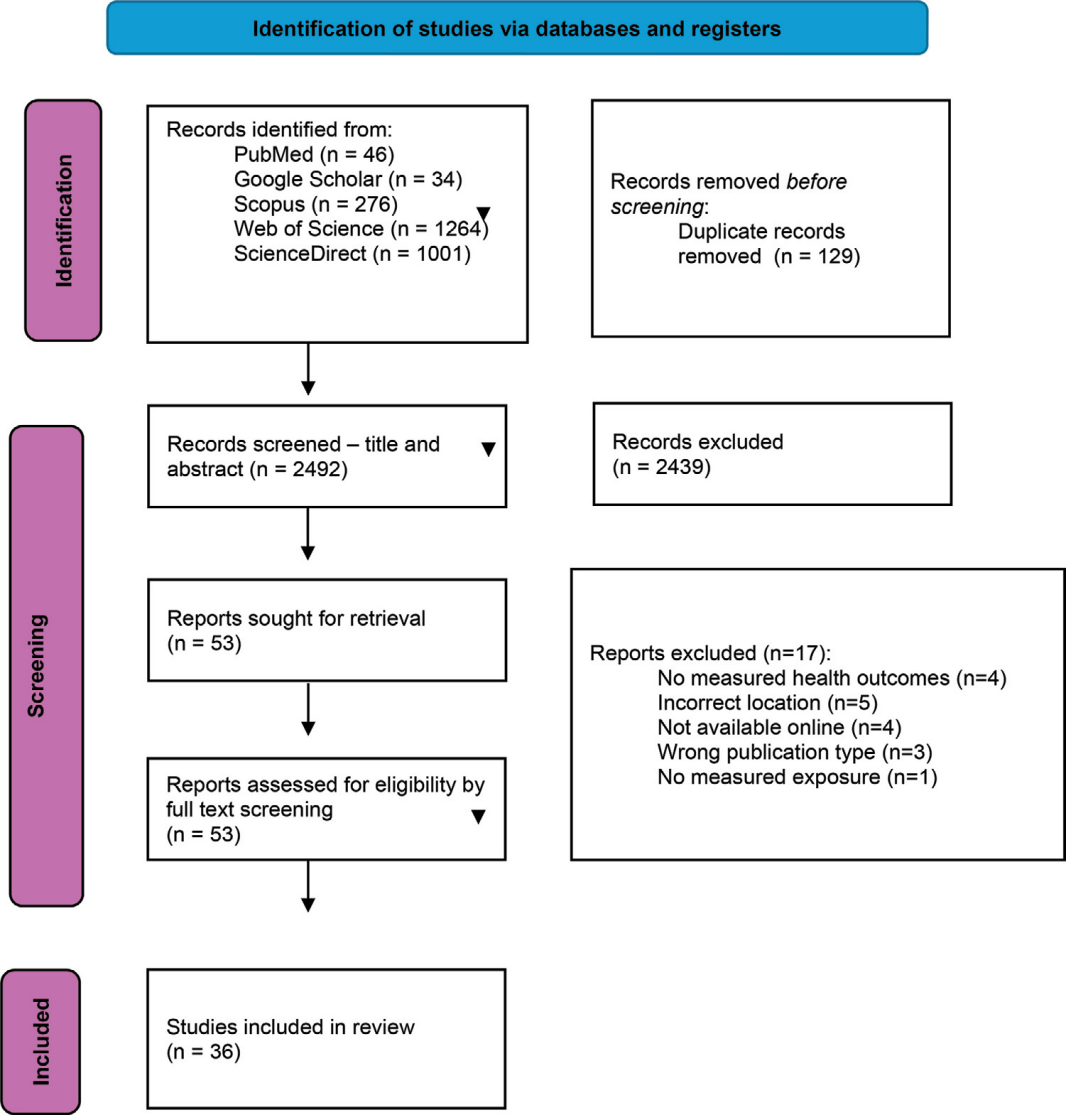
PRISMA diagram for this study.

We included 36 studies in the final review (Figure 2). Study years spanned 2006 to 2024 (using the minimum and maximum years reported, including ranges). The full study findings are included in Supplementary File F1 and summarized in Table 3. In terms of study designs, the included studies were dominated by a small number of recurrent designs, with time-series and cross-sectional approaches most common, and a smaller number of modeling/forecasting and qualitative/occupational studies. Since we focused on South African studies, reported regions skew toward South Africa and Southern Africa, with additional multi-country and city-level analyses; tokenized region strings indicate the most frequent location mentions that include South Africa, Cape Town, Gauteng/Johannesburg, and broader “Southern Africa/Africa” groupings.

**Table 3:** Summary of descriptive findings of the 36 included studies in this scoping review.

FIRST AUTHOR, YEAR OF PUBLICATION	STUDY DESIGN, PLACE	METHODS APPLIED IN THE STUDY	FINDINGS
Brimicombe, 2024 [22]	Observational, Africa	Pooled time-series analysis Risk ratio for mortality rates	The influence of extreme heat on mortality risk in children under five years varies by age group, region, and season. There was a significant increase in mortality risk for children in eastern Africa, Senegal, and The Gambia. The study highlights the importance of considering seasonal and regional variations when assessing heat exposure effects.
Buhler, 2022 [23]	Observational, Limpopo	Distributed lag, non-linear model	Both warm and cold apparent temperatures are associated with an increase in cardiovascular disease hospital admissions, with a cumulative effect over 21 days. The effect of warm temperatures is immediate and short-lived, lasting two to four days, while the effect of cold temperatures is more inconsistent. Cold temperatures account for a larger fraction (8.5%) of CVD admissions compared to warm temperatures (1.1%).
Burkart, 2021 [24]	Observational, global	Estimated population attributable fractions for high and low temperatures	The study estimated that 1.69 million deaths were attributable to non-optimal temperature globally in 2019. Cold-attributable mortality generally exceeded heat-attributable mortality, with regional variations in burden. High heat-attributable burdens were observed in South and Southeast Asia, sub-Saharan Africa and North Africa, and the Middle East, while high cold-attributable burdens were seen in Eastern and Central Europe and Central Asia.
Gates, 2019 [25]	Observational, South Africa	Time-stratified case-crossover	A 1°C increase in same-day maximum temperature was associated with a 1.5% increase in definite homicides and a 1.2% increase in total homicides. Significant positive associations were observed across different temperature metrics and lags. The temperature–homicide association was linear, with no clear non-linearities.
Hugo, 2023 [19]	Observational, mixed methods, Pretoria	Indoor monitoring Local weather data Surveys, reflexive diaries	Informal dwellings in Melusi, Tshwane, South Africa, perform poorly due to endogenous factors, leading to extreme heat stress conditions for 6 to 10 h daily during peak summer. The dwellings’ spatial and material conditions are similar, with limited thermal control strategies, resulting in high indoor temperatures. The study highlights the need for significant adaptations to mitigate heat stress but notes that such adaptations may be socially and economically unfeasible.
Kapwata, 2022 [26]	Observational, South Africa	Diurnal temperature range threshold defined heatwave Calculated hot days per district	The highest frequency of heatwaves in South Africa occurred during the austral summer, with 150 events out of 270 from 2014 to 2019. Future climate projections indicate an 80%–87% increase in heatwave events during summer months from 2020 to 2039. The study highlights the need for targeted heat-health adaptation strategies by identifying provinces and towns with intense, long-lasting heatwaves.
Kapwata, 2021 [27]	Observational, Limpopo	Wavelet transform analysis	The study found that changes in air quality precondition the prevalence of pneumonia, with a time delay of 10 to 15 days. Malaria in South Africa is a multivariate event initiated by the co-occurrence of high temperature and rainfall, with a time delay of 30 days. The findings have relevance for early warning system development and climate change adaptation planning to protect human health and well-being.
Kapwata, 2024 [28]	Observational, South Africa	Distributed lag, non-linear modeling	Maximum temperature was identified as the most statistically significant predictor of all-cause mortality in 40 out of 52 districts in South Africa. The spatial distribution of maximum temperature thresholds varied by climate zone, with higher thresholds in hotter regions indicating population adaptation to local climate. The study recommends using maximum temperature as a key metric for heat-health warning systems due to its strong association with mortality and adaptability to local climate conditions.
Lokotola, 2020 [29]	Case-crossover, Cape Town	Time-stratified case-crossover Distributed lag, non-linear modeling	The 15–64-year-old group was more at risk for CVD hospitalization with increasing air pollution levels on warm and cold days compared to other age groups. Females were more at risk from PM_10_, while males were more vulnerable to NO_2_ and SO_2_. Temperature was identified as a modifier of the effects of air pollution on CVD hospital admissions, with varying impacts across different population subgroups.
Mathee, 2009 [30]	Qualitative, Johannesburg and Upington	Focus group discussions Interviews Thematic analysis	Workers in Upington experienced heat-related health effects such as sunburn, sleeplessness, irritability, and exhaustion, which affected their work levels and output. Few measures were taken by employers to protect workers’ health or improve their comfort. The study highlights the need for further research to quantify the effects of outdoor work on workers due to its public health importance.
McElroy, 2021 [31]	Pooled time-stratified case-crossover, global	Pooled time-stratified case-crossover design DLNM	Higher maximum temperatures and smaller diurnal temperature ranges in the week before birth increased the risk of preterm birth and stillbirth. The risk of preterm birth increased with exposure to extreme heat within seven days before birth, indicating an immediate effect. A temperature range of 20°C–30°C was identified where the risk of stillbirth is heightened, indicating a threshold beyond which the risk increases.
McMichael, 2025 [32]	Observational, global	Modeling	Temperature is associated with daily mortality in cities across different climates, with increased risk at both high- and low-temperature extremes. Cities with hotter summers have higher heat thresholds, reflecting population adaptation to local climate conditions. Populations in low- and middle-income countries are adversely affected by high temperatures and may be especially vulnerable to future climate change impacts.
Mfamadi, 2023 [33]	Cross-sectional, Johannesburg	Temperature measurements Questionnaire	Median indoor apparent temperature in shipping container units ranged from 6°C to 42°C, indicating potential heat-health risks. All units experienced heat-health risks, with some exceeding danger thresholds, and participants reported symptoms like headaches and fatigue. Insulation may not be adequate for maintaining thermal comfort, suggesting a need for alternative solutions to mitigate heat-related health impacts.
Moodley, 2024 [34]	Observational, KwaZulu-Natal	Population-based cohort analysis	Each additional hot day during the month preceding conception was associated with 26% higher odds of miscarriage. No significant association was found between heat exposure during the week preceding the outcome and miscarriage. The relationship between heat exposure and miscarriage was J-shaped, with most miscarriages occurring within the first four weeks post-conception.
Manyuchi, 2022 [10]	Observational, Agincourt	Interviews	Heat exposure has serious effects on morbidity, particularly affecting vulnerable populations such as the elderly and children. Future health awareness campaigns are essential to reduce vulnerability, morbidity, and mortality. The study provides location-specific data for an area expected to experience significant heat stress, underscoring the need for heat-health interventions.
Mwase, 2022 [35]	Case-crossover, Vaal Triangle Air Pollution Priority Area	Time-stratified case-crossover	The association between respiratory disease hospitalizations and air pollution in the Vaal Triangle Air Pollution Priority Area is modified by apparent temperature (*T*_app_). Moderate *T*_app_ levels worsen the effects of PM_2.5_, PM_10_, SO_2_, and BC, while high *T*_app_ levels are more associated with increased effects of NO_2_ and O_3_. The elderly and females are more vulnerable to air pollution, especially on days with moderate *T*_app_ levels.
Naicker, 2017 [36]	Cross-sectional, Johannesburg	Indoor monitoring Questionnaire	There was a significant difference in monthly mean indoor temperature between different housing types, with low-cost government-built houses and informal settlement houses experiencing the greatest variation. These housing types were warmer than outdoor temperatures by 4°C–5°C, indicating a higher risk for indoor temperature extremes. The presence of ceilings significantly affected indoor temperature stability, with dwellings without ceilings being more sensitive to ambient temperature changes.
Ncongwane, 2021 [37]	Observational, multiple	Heat stress indicators using apparent temperature	The study found positive trends in heat stress in 88% of the weather stations, with approximately 47% being statistically significant at a 5% level. High annual climatological median values (>32°C) were observed at 42 stations, mostly in low-altitude regions along coastlines. The study identified regions in South Africa susceptible to heat stress, particularly low-altitude areas in provinces like MP, KZN, LP, WC, NC, and EC.
Nhamo, 2025 [38]	Observational, South Africa	In-depth interviews Thematic analysis	Droughts, extreme heat, wildfires, and floods are the most prevalent climate-induced weather extremes in South African national parks. These weather extremes lead to loss of biodiversity, wildlife stress, damage to infrastructure, reduced tourist arrivals, and loss of revenues. Park managers adopt strategies such as building back better infrastructure, creating fire breaks, and implementing water conservation measures to combat these impacts.
Olutola, 2023 [39]	Observational, Cape Town	Time-stratified case-crossover Effect modification by temperature	The elderly and females are more vulnerable to air pollutants, especially at high and moderate apparent temperature levels. NO_2_ has stronger effects on CVD mortality compared to PM_10_ and SO_2_. Harvesting effects were observed at longer lags, indicating decreased risk over time. The results can be used to develop an early warning system for CVD mortality in Cape Town.
Orlov, 2024 [40]	Observational, global	Model simulations	The study found that under the RCP1.9 scenario, there is a reduction in total excess mortality by the end of the century compared to 1980–1989, primarily due to a decrease in cold-related deaths. The results indicate limited net negative impacts of temperature-related mortality under RCP1.9, with some regions experiencing a marginal net positive benefit. The study highlights substantial differences in temperature responses across the three Earth system models used.
Part, 2022 [41]	Observational, Johannesburg	Time-to-event	High temperatures in early pregnancy, particularly during the third and fourth weeks, increase the risk of pre-eclampsia, eclampsia, and HELLP syndrome. Low temperatures during the third trimester are associated with an increased risk of high blood pressure, hypertension, and gestational hypertension. High temperatures in early pregnancy likely affect placental development, increasing the risk of severe hypertensive disorders.
Rother, 2019 [42]	Observational, Western Cape	Focus group discussions	Forestry workers in South Africa experience health impacts from heat and sun exposure, using local coping mechanisms like wearing ochre for protection. The study identified gaps in current protective measures and suggested improvements such as providing individual water bottles, sunscreen, and flexible work hours. Workers initially did not differentiate between sun and heat exposure, but through discussions, they began to understand the differences.
Scovronick, 2012 [43]	Observational, Western and Eastern Cape	Distributed lag, non-linear model	Future mortality burdens would be lower under a policy scenario prioritizing the replacement of informal housing over traditional dwellings. A maximum protection scenario with formal wealthy housing could reduce temperature-related mortality by over 50%, approximately 5000 deaths annually. Well-planned housing policies can reduce the future burden of temperature-related mortality, emphasizing the need to improve low-cost housing thermal performance.
Scovronick, 2018 [44]	Observational, South Africa	Distributed lag, non-linear model	The study found an association between daily maximum temperature and mortality, with increased risks at both cold and hot temperatures. Overall, 3.4% of deaths in South Africa were attributed to non-optimum temperatures, with the majority due to cold temperatures. The strongest associations were observed in the youngest and oldest age groups, as well as for cardiorespiratory causes.
Teare, 2020 [45]	Observational, Port Elizabeth, and Johannesburg	Temperature measurements Questionnaire	Upper respiratory tract symptoms were significantly associated with reports of high levels of indoor dust. Lower respiratory tract symptoms were significantly associated with low income, overcrowding, and having a young child in the household.
Wen, 2024 [46]	Observational, global	Quasi-Poisson time-series Multi-level analysis	Intra-day temperature variability (TV) is associated with a higher mortality risk than inter-day TV for all-cause, cardiovascular, and respiratory mortality. The mortality risk increases by 0.59% for all-cause, 0.64% for cardiovascular, and 0.65% for respiratory mortality per IQR increase in intra-day TV 0–7. Intra-day TV accounts for 1.45% of all-cause deaths, while inter-day TV accounts for 0.35%.
Wichmann, 2017 [47]	Case-crossover, Cape Town, Durban, and Johannesburg	Time-stratified Meta-analysis	A significant increase in mortality was observed when apparent temperature exceeded city-specific thresholds. The elderly (≥ 65 years) were more at risk, with a significant increase in mortality per IQR increase in *T*_app_. The observed risks are similar to those in other countries, indicating that South Africa’s population is not more vulnerable to heat-related mortality.
Wright, 2017 [48]	Observational, Limpopo	Indoor monitoring Questionnaire	Mean monthly indoor temperatures in clinics were higher during summer months, with the highest mean temperature of 31.4 ± 2.7°C recorded in February 2016. Indoor temperatures were 2°C–4°C higher than outdoor temperatures on average, with apparent temperatures suggesting discomfort and potential health risks. The study supports the need for controlled temperatures, reduced waiting times, and other measures to mitigate heat-related health impacts in clinics.
Wright, 2017 [49]	Observational, Durban	Cross-sectional questionnaire	Glare from the sun and excessive sweating were commonly reported sun-related health symptoms among informal workers. The use of protective clothing was more prevalent among those who perceived sun exposure as a hazard. Informal workers implemented their own protective measures, such as portable shade and protective clothing, due to high sun exposure.
Wright, 2019 [50]	Observational, Johannesburg	Cross-sectional questionnaire	Individuals with hypertension and those over 60 years old are more likely to experience heat-related health effects. Living in government-sponsored detached housing and houses with asbestos roofs increases the risk of heat-related health effects. Targeted awareness campaigns for individuals with pre-existing diseases and the elderly, and improvements in housing infrastructure, are recommended to mitigate heat-related health impacts.
Wright, 2025 [51]	Observational, Durban	Mixed methods Temperature monitoring Questionnaire	Temperatures inside minibus taxis reached up to 39°C, significantly warmer than outdoors, and were above 27°C for extended periods, posing a threat to human health. Taxi drivers reported feeling hot and used water to cool down, indicating subjective heat stress. The study suggests that high temperatures could lead to dehydration and heat-related illnesses among drivers, necessitating mitigation strategies due to climate change.
Wu, 2022 [52]	Two-stage time series, observational, global	Temperature variability calculation Attributable fraction	The study found that increased temperature variability (TV) is associated with higher heat-related mortality risks, with attributable fractions ranging from 0.70% to 2.73% for different quartiles of TV. TV has a significant modification effect on the heat-mortality association, leading to a higher mortality burden with increased TV. The combination of heat exposure and high TV significantly increases mortality risk during the warm season, with disparate geographical variations in the impact of TV.
Wu, 2024 [53]	Observational, global	Two-stage time series Multilevel meta-analysis	The study observed a consistent decrease in mortality risk as the frequency of temperature increases, indicating adaptation to local climate through frequent exposure. The average increase in mortality risk from the 10th to the 100th percentile of normalized frequency was 13%, with significant regional differences. The increase in mortality risk varied by temperature component, with extreme cold showing the highest increase, followed by extreme hot, moderate cold, and moderate hot.
Zhu, 2023 [54]	Cross-sectional, sub-Saharan Africa	Predictive modeling	The study found that each 1°C increment in annual temperature was associated with increased odds of anemia in children. The burden of childhood anemia attributable to climate warming is projected to increase significantly under a high-emission scenario by the end of the 21st century. Regional disparities were observed, with Central Africa expected to bear the highest burden of childhood anemia due to global warming.
Zhao, 2024 [55]	Observational, global	Modeling framework, distributed lag non-linear model	Heatwave-related excess deaths accounted for 0.94% of global deaths during the warm seasons of 1990 to 2019, equating to 236 deaths per 10 million residents. The global heatwave-related excess death ratio remained relatively constant, while the excess death rate declined by 7.2% per decade. The findings indicate the potential benefit of governmental actions to enhance health sector adaptation and resilience, accounting for inequalities across communities.

Frequently used methods included epidemiological time series linking meteorological exposure (temperature/heat indices) and/or air pollutants (e.g., PM_2.5_, PM_10_, NO_2_, O_3_) with health outcomes; remote-sensing, reanalysis, or station data for exposure assessment; and regression or distributed lag models. Among the 36 included studies, the most common findings involved heat and air pollution associations with morbidity (including clinic visits, hospital admissions) and mortality, with elevated risks in vulnerable populations (namely, low-income communities, children, and older adults) and outdoor workers exposed to high ambient or indoor temperatures. Most studies did report study limitations, and these limitations clustered around exposure measurement error, spatial representativeness (limited monitoring), outcome data completeness, and generalizability.

Of the included studies, 31 mentioned policy recommendations that frequently emphasize heat-health action plans, early-warning systems, urban/occupational heat mitigation, and air-quality management. Several future research priorities were suggested and included improved exposure data (by using higher spatial/temporal resolution), evaluation of proposed and implemented interventions, and better characterization of vulnerability factors and sector-specific risks.

### Results of the benchmarking exercise: Recommendations for governance and implementation

We identified three provincial exemplars for our benchmarking exercise from India, Australia, and Pakistan and cross-checked them against the new synthesis report published by the United Nations Office for Disaster Risk Reduction [56], the World Meteorological Organization, the Global Heat Health Information Network, and the Nicholas Institute titled “An assessment of heat action plans: Global standards, good practices and partnerships” to anchor the benchmarking, and these findings are described below [56].

**State Action Plan for Climate Change and Human Health for Maharashtra State, India:** This HAP is led by the State Disaster Management Authority (SDMA) and entails a statewide plan naming high-risk regions (Vidarbha, Marathwada, Khandesh) [57]. The plan uses India’s Meteorological Department’s definitions for heatwaves: heatwave = +4.5°C–6.4°C above normal and severe heatwave > 6.4°C [57]. In terms of operations, health, disaster management, municipalities, and line departments, all have defined actions, and the National Centre for Disease Control, Government of India, provides health-sector implementation guidance and surveillance linkages [58].

**Heatwave Plan for Victoria, Australia:** This plan [59] is health-led and integrated with emergency management. It advocates for district-specific “heat-health temperature thresholds” (based on forecast average temperature) that are aligned with emergency/fire districts while being simple, transparent, and locally tuned. Pre-scripted communications to services and the public are activated by local government activation to prepare health-system surge readiness.

**Khyber Pakhtunkhwa Heat Wave Action Plan, Pakistan:** The Provincial Disaster Management Authority (PDMA-KP) Khyber Pakhtunkhwa Heat Wave Action Plan 2022 contains vital information on early warning and coordination, declaration protocols, surveillance, and role matrices [60]. Details are provided for agency roles and community measures, for example, cooling/“heat stabilization” camps mobilized via multi-partner deployment, illustrating scalable low- and middle-income country tactics (see, for example, [61]).

Learnings from India, Australia, and Pakistan include provincial-wide risk mapping; graded triggers tied to South African Weather Service (SAWS) forecasts with templated alerts that are part of an automatic notification chain that fires when thresholds are crossed; and department-specific checklists that escalate responses from advisory to warning to emergency levels. Low-cost, rapidly deployable cooling points (such as clinics, libraries, shopping malls, and taxi ranks) are essential and scalable with sufficient resources. These elements are also emphasized in the UNDRR report [56] that urges for standard definitions, a clear lead agency, multi-sector coordination, multi-sector governance, stronger, robust early warning systems, evaluation, and public–private partnerships. Some of the strengths we recommend that Gauteng adopt are elaborated in Table 4.

**Table 4:** Benchmark matrix of HAP elements that Gauteng Province should consider adopting for their own HAP.

	REVIEWED LOCATIONS			
DIMENSION AND LOCATION INFORMATION	MAHARASHTRA	VICTORIA	KHYBER PAKHTUNKHWA	IMPLICATION FOR GAUTENG
**Lead and roles**	State Disaster Management Plan with line-department playbooks	Health-led and emergency management integration	Provincial Department Management Authority declaration and Emergency Operations Centre protocols	Gauteng Department of Health lead; Provincial Disaster Management Centre co-lead; formal memorandum of understandings with metros and SAWS
**Triggers**	Indian Meteorological Department anomaly-based (graded)	District-specific forecast mean thresholds	Heat index and declaration steps	District-specific thresholds with SAWS, published in advance
**Early warning**	Statewide cascade; media/toolkits	Pre-scripted alerts to sectors and public	Provincial Department Management Authority alerts; inter-agency activation	WhatsApp/SMS and radio cascade; signage at clinics/taxi ranks
**Health system**	Heat illness surveillance and facility actions	Surge plans; checklists for services	Standard Operating Procedures across health and rescue	Emergency Department triage, IV fluids, electrolytes stock, staff rostering for hot days
**Community measures**	Public advice, hydration, shade	Local government activation and outreach	Cooling camps during heat events	Pop-up cooling points/water points
**Equity**	Vulnerable district targeting	Targeted messaging to services	Community-level access points	Prioritize informal settlements, elderly, outdoor workers
**Monitoring and evaluation**	State Guidance via National Centre for Disease Control	Annual plan refresh	Post-season reviews implied	After-action review each season; track heat-related illnesses, mortality, alert reach
**Long-term**	Heat-resilient infrastructure and urban planning linkages (per global guidance)	Integrated with broader risk management	Standard Operating Procedures and risk analysis baseline	Trees, cool roofs, sharing at clinics and schools, aligning with the Sustainable Development Goals and Sendai Framework

Presented in full in Supplementary Material S1, HAPs across France, England, the Netherlands, British Columbia, Japan, and Italy share three core activities—warning systems, institutional frameworks, and measures for vulnerable populations—with highly transferable lessons being early warning systems, targeted vulnerable group support, and multi-channel communication that form a transferability framework based on meteorological triggers, institutional roles, and community engagement for application in other countries.

## Discussion

This review provides consolidated evidence on heat-related health impacts in Gauteng, contextualized within South Africa and benchmarked against international and/or subnational HAPs. Across 36 studies, evidence emerged that high ambient temperatures, heatwaves, and diurnal variability are associated with increased risks of morbidity and mortality, particularly among vulnerable groups such as children, older adults, and residents of informal settlements [23, 26, 28, 39, 44]. Indoor heat exposures, whether in housing, clinics, or transport, were consistently above comfort and safety thresholds, underscoring the importance of interventions beyond outdoor heat warnings [30, 33, 36, 42].

The evidence from these studies has provided insights on areas to prioritize in the implementation of heat-warning systems. First, maximum temperature was consistently identified as a strong predictor of mortality and morbidity, suggesting it should be prioritized in local heat warning systems [26]. Second, the modifying role of air pollution on heat-health outcomes indicates that Gauteng’s HAP should integrate air-quality management with heat preparedness [29]. Third, adaptation strategies will need to address the structural inequities that amplify vulnerability, including inadequate housing insulation [43], limited access to cooling [62] and occupational exposures [30, 42].

The benchmarking exercise highlighted transferable elements for Gauteng. Victoria’s district-based thresholds are relevant given Gauteng’s diverse microclimates, while Maharashtra’s graded triggers and departmental clarity could strengthen intersectoral coordination across health, infrastructure, and municipal actors. Khyber Pakhtunkhwa’s low-cost cooling camp model demonstrates scalable interventions for resource-constrained contexts, which could be adapted for clinics and taxi ranks in Gauteng. Overall, there is a need for clearly defined lead agencies, routine evaluation of action plans, and accessible communication strategies targeting vulnerable groups.

### Strengths and limitations

The limitations of the scoping review include potential publication and selection biases. The review may have missed some new articles since we concluded our search in mid-2025, while different or more refined search terms may have uncovered different articles that we missed. Our search was limited to the English language, and articles from other languages, such as from authors writing in Afrikaans, were not included. For the benchmarking, we selected one high-income country and two low-income countries as illustrative examples, and had we chosen other countries, we may have had different findings.

## Conclusions

Extreme heat is a global public health concern due to the rising burden of diseases and increased risk of mortality associated with exposure to high temperatures. Therefore, HAPs, such as heat early warning systems, are crucial to reducing the human and financial costs of heat events. Learnings from India, Australia, and Pakistan show that city-specific heat alerts are effective in promoting climate-resilient cities and preventing loss of life. Given the range of adverse health outcomes linked to high temperatures in Gauteng, cities within the province could benefit significantly from the implementation of localized early heat warning systems to ensure appropriate responses to hot weather. Furthermore, drawing on the knowledge gained from countries with operational systems will strengthen the effectiveness of such heat adaptations.

## Additional File

The additional file for this article can be found as follows:

Supplementary material. S1. Analysis of national scale heat action plans.
