# Supplementary material for: Temperature-Related Health Impacts: A Scoping Review and Benchmarking Exercise to Inform a Heat Action Plan
Source: Ann Glob Health. 2026 Jan 22;92(1):8. doi: 10.5334/aogh.5016 (PMC12829453; doi:10.5334/aogh.5016)
Supplement: Supplementary material. — S1. Analysis of national scale heat action plans. [file agh-92-1-5016-s1.pdf]

Supplementary Material

S1. Analysis of national scale heat action plans

Heat action plans in France, England, the Netherlands, British Columbia, Japan, and Italy rely on three core activities: warning systems, institutional frameworks, and measures for vulnerable populations (See Table S1, S2). Studies from Italy and France describe national programs that use threshold-based warnings, registries, and active surveillance to reach at-risk groups. In Japan, a ministry-led approach employs Wet Bulb Globe Temperature metrics and local government support to trigger alerts and guide community outreach. In England, plans associated with the National Health Service suffer from fragmented communication and limited frontline integration, while work in British Columbia emphasizes interagency partnerships, including indigenous and community organizations. Dutch examples stress institutional protocols in care settings and the need for locally adapted research. Several evaluated lesson categories appear directly transferable to other settings (Table S3). Papers indicate that early warning systems, targeted support for vulnerable groups, and multi-channel communication strategies hold high transferability. Cross-sectoral governance and systematic evaluation receive moderate transferability ratings, whereas integration of mental health measures appears less supported. These concrete activities and documented lessons offer a transferability framework for heat action plan development based on clear meteorological triggers, defined institutional roles, and tailored community engagement.

Table S1. Characteristics of included studies.

Study	Country/Region	Study Focus	Heat Action Plan Components	Methodology
de 'Donato et al., 2018	Italy	Temporal change in heat-related mortality post-national heat plan	No mention found in abstract	Time series analysis; distributed lag non-linear models; qualitative survey
Signer et al., 2023	Canada (British Columbia, Victoria)	Community resilience and adaptation post-2021 heat dome	Vulnerable population identification; community engagement	Case study
Salagnac, 2007	France	Lessons from 2003 heatwave	No mention found in abstract	No mention found
Deegan et al., 2022	Canada (British Columbia)	Heat Alert and Response System (HARS) development in rural community	Early warning, meteorological triggers, communication, engagement, vulnerable groups, healthcare mobilization	Qualitative; mixed methods
Michelozzi et al., 2010	Italy	National program for heat-health prevention	Early warning, meteorological triggers, communication, engagement, vulnerable groups, healthcare mobilization	Mixed methods
Pascal et al., 2021	France	Challenges to heat warning systems	Early warning, meteorological triggers, communication, engagement, vulnerable groups, healthcare mobilization, evaluation	Mixed methods

Kunst and Britstra, 2013	Netherlands	Evaluation of Dutch national heat plan in care institutions	Vulnerable group identification, cooling measures, protocols, staff training	Qualitative with quantitative data
Hong et al., 2022	France, South Korea	Comparative review of Paris and Seoul Heat Action Plans	Early warning, vulnerable group support, communication, engagement	Comparative case study
Heudorf and Schade, 2014	Germany	Mortality in Frankfurt post-Heat-Health Action Plan	Early warning, communication, engagement, vulnerable groups, healthcare mobilization	Comparative case study; quantitative
Scortichini et al., 2018	Italy	Temporal variation in heat effect post-plan	No mention found in abstract	Quantitative (distributed lag non-linear models)
Brooks et al., 2024	England	Health system resilience during 2019 heatwave	No mention found in abstract	Qualitative
Boyson et al., 2014	England	Evaluation of National Heatwave Plan in hospital	No mention found in abstract	Qualitative
Vachat et al., 2004	France	Adaptation to heatwaves post-2003	Early warning, communication, French National Heatwave Plan (Plan Canicule)	No mention found
Pavanello et al., 'Mortality, Temperature, and Public Adaptation Policy'	Italy	Impact of national program on mortality	Public awareness, warning systems, hospital protocols	Comparative case; quasi-experimental
Tonouchi, 2013	Japan	Heat Disorder Information System	Early warning, meteorological triggers, communication, engagement, vulnerable groups, healthcare mobilization	Qualitative

Pascal et al., 2006	France	Development of Heat-Health Warning and Watch System (HHWWS)	Early warning, meteorological triggers, communication, engagement, healthcare mobilization	Mixed methods
McLean et al., 2018	Canada (British Columbia)	Establishing heat alert thresholds	Early warning, meteorological triggers, communication, engagement, vulnerable groups, healthcare mobilization	Systematic review; mixed methods
Bittner et al., 2013	Not specified in abstract	Policy development/implementation in Europe	Indoor heat reduction, urban planning, surveillance	Qualitative
McLean et al., 'Establishing Appropriate Hot Weather Alerting Criteria'	Canada	Hot weather alerting criteria	Early warning, meteorological triggers, local adaptation	Quantitative (time-series)
Howarth et al., 2025	United Kingdom	Coping vs. resilience in 2022 heatwave	No mention found in abstract	Qualitative
Sario et al., 2011	Italy	Heat-Health Warning and Watch System models and prevention	Early warning, communication, vulnerable groups, prevention activities	Mixed methods
Berry et al., 2014	Canada	Heat Alert and Response System in urban/rural communities	Mobilization, alert protocol, response plan, communication, evaluation, prevention	Comparative case; mixed methods
Dwyer et al., 2022	Europe, India, Australia	Evaluation methods for Heat Action Plans	Heat Warning System, Heat-Health Action Plan, universal heat index	Systematic review
Bittner et al., 2014	Europe (18 countries)	Assessment of Heat Action Plans in WHO Europe	8 core elements (lead body, alert,	Systematic review

			information, indoor heat, vulnerable groups, preparedness, urban planning, surveillance)	

Table S2. Heat Action Plan Development Activities.

Study	Country/Region	Warning Systems	Institutional Framework	Vulnerable Population Measures
de ‘Donato et al., 2018	Italy	No mention found	National heat plan, survey of measures	No mention found in abstract
Signer et al., 2023	Canada (British Columbia, Victoria)	No mention found	Municipal, provincial, and health authority collaboration	Strategies for vulnerable groups, community engagement
Salagnac, 2007	France	No mention found	No mention found	No mention found in abstract
Deegan et al., 2022	Canada (British Columbia)	Heat alert thresholds, British Columbia Centre for Disease Control triggers	Health authority, municipal, First Nations, community organizations	Identification and engagement of vulnerable groups
Michelozzi et al., 2010	Italy	City-specific Heat-Health Warning and Watch System	National program, local adaptation	Registries, active surveillance, targeted prevention
Pascal et al., 2021	France	Joint Santé publique France/Météo-France system	National plan, feedback loops	Registries, call centers, management plans
Kunst and Britstra, 2013	Netherlands	No mention found	National heat plan, institutional protocols	Protocols, cooling, staff training for vulnerable groups
Hong et al., 2022	France, South Korea	Early warning systems	National plans, urban/social policy integration	Support for vulnerable, infrastructure expansion
Heudorf and Schade, 2014	Germany	National warning system	Local authorities, public health services	Targeted information, inspections, visits
Scortichini et al., 2018	Italy	No mention found	National heat plan	No mention found in abstract
Brooks et al., 2024	England	No mention found	National Health Service, hospital-based	No mention found in abstract
Boyson et al., 2014	England	No mention found	Hospital management	No mention found in abstract

Vachat et al., 2004	France	Alert system, vigilance map	French National Heatwave Plan (Plan Canicule)	No mention found in abstract
Pavanello et al.	Italy	Warning systems	National program, hospital protocols	No mention found in abstract
Tonouchi, 2013	Japan	Wet Bulb Globe Temperature-based system	Ministry of Environment, local governments	Focus on elderly, seminars, guidebooks
Pascal et al., 2006	France	Heat-Health Warning and Watch System, 4-level response	National action plan, interagency	Real-time data, not explicit
McLean et al., 2018	Canada (British Columbia)	High-low-high thresholds	British Columbia Centre for Disease Control, Environment and Climate Change Canada, Health Canada	Sub-analyses for risk groups

Table S3. Transferability Framework Application.

**Lesson Category**	**Countries of Origin**	**Transferability Level**	**Implementation Requirements**
Early warning systems	France, Italy, Japan, Canada, Netherlands	High	Meteorological data, health surveillance, local adaptation
Vulnerable population targeting	France, Italy, Netherlands, Canada, Japan	High	Registries, outreach, tailored communication, staff training
Cross-sectoral governance	France, Italy, Netherlands, Canada, United Kingdom	Moderate	Interagency agreements, clear roles, stakeholder engagement
Evaluation and surveillance	France, Italy, Europe	Moderate	Real-time data, feedback loops, resource allocation
Communication strategies	All	High	Multi-channel messaging, media engagement, tailored content
Local adaptation	All	High	Needs assessment, resource mapping, community engagement
Mental health integration	Global (few plans)	Low	Evidence base, resource allocation, tailored interventions

Supplementary material references

Filippo Pavanello, Giulia Valenti, Irene Mammi, and Francesco Vona. "Mortality, Temperature, and Public Adaptation Policy: Evidence from Italy *," n.d.

A. Casanueva, Annkatrin Burgstall, S. Kotlarski, A. Messeri, M. Morabito, A. Flouris, L. Nybo, C. Spirig, and C. Schwierz. "Overview of Existing Heat-Health Warning Systems in Europe." *International Journal of Environmental Research and Public Health*, 2019.

A. Kunst, and R. Britstra. "Implementation Evaluation of the Dutch National Heat Plan Among Long-Term Care Institutions in Amsterdam: A Cross-Sectional Study." *BMC Health Services Research*, 2013.

Allison Stewart-Ruano, Raenita Spriggs, E. Lawrance, Alessandro Massazza, Alexandra Czerniewska, Alejandro Sáez Reale, Joy Shumake-Guillemot, Katherine M. Keyes, Yoshira Ornelas Van Horne, and Robbie Parks. "A Critical Gap in Addressing Mental Health in Heat-Health Action Plans Worldwide." *Current Environmental Health Reports*, 2025.

C. Brimicombe, James J. Porter, C. Napoli, F. Pappenberger, R. Cornforth, C. Petty, and H. Cloke. "Heatwaves: An Invisible Risk in UK Policy and Research." *Environmental Science and Policy*, 2021.

C. Howarth, Niall McLoughlin, Sara Mehryar, Ellie Murtagh, Andrea Armstrong, and James Porter. "Boiling Point: Short-Term Coping with Heatwaves in the UK Is Not Enough." *Environmental Research Letters*, 2025.

Chris Boyson, Sarah Taylor, and Lisa Page. "The National Heatwave Plan – A Brief Evaluation of Issues for Frontline Health Staff." *PLOS Currents*, 2014.

Emily J. Tetzlaff, Nicholas Goulet, Melissa Gorman, Gregory R. A. Richardson, Paddy M. Enright, Robert D. Meade, and Glen P. Kenny. “Hot Topic: A Systematic Review and Content Analysis of Heat-Related Messages During the 2021 Heat Dome in Canada.” *Journal of Public Health Management and Practice*, 2023.

F. de'Donato, M. Scortichini, M. D. Sario, A. D. Martino, and P. Michelozzi. “Temporal Variation in the Effect of Heat and the Role of the Italian Heat Prevention Plan.” *Public Health*, 2018.

F. Matthies, and B. Menne. “Prevention and Management of Health Hazards Related to Heatwaves.” *International Journal of Circumpolar Health*, 2009.

Fariha Hasan, Shayan Marsia, K. Patel, Priyanka Agrawal, and J. Razzak. “Effective Community-Based Interventions for the Prevention and Management of Heat-Related Illnesses: A Scoping Review.” *International Journal of Environmental Research and Public Health*, 2021.

Franziska Baack, Johannes Halman, Joanne Vinke-de Kruijf, G. Özerol, and Stefan M. M. Kuks. “Dutch Municipalities Tackling Climate Change Adaptation to Heat Stress Through Mainstreaming Across Sectors.” *Environmental Science &Amp; Policy*, 2024.

G. S. Martinez, Chisato Imai, and Kanako Masumo. “Local Heat Stroke Prevention Plans in Japan: Characteristics and Elements for Public Health Adaptation to Climate Change.” *International Journal of Environmental Research and Public Health*, 2011.

G. S. Martinez, V. Kendrovski, M. A. Salazar, F. de ’Donato, and M. Boeckmann. “Heat-Health Action Planning in the WHO/Europe: Status and Policy Implications.” *Environmental Research*, 2022.

G. Sanchez Martinez, V. Kendrovski, and F. de 'Donato. "Heat and Health Prevention in European Region Under a Changing Climate: The Urgent Need for a Faster Roll-Out of Heat-Health Action Plans (HHAPs)." *ISEE Conference Abstracts*, 2022.

H. A. Grewe, S. Heckenhahn, and B. Blättner. "[Health Protection During Heat Waves: European Recommendations and Experience in Hesse]." *Zeitschrift Für Gerontologie Und Geriatrie (Print)*, 2014.

H. Deegan, J. Green, Sylvia El Kurdi, Michelle Allen, and S. Pollock. "Development and Implementation of a Heat Alert and Response System in Rural British Columbia." *Canadian Journal of Public Health*, 2022.

I. Dwyer, S. J. E. Barry, I. Megiddo, and C. White. "Evaluations of Heat Action Plans for Reducing the Health Impacts of Extreme Heat: Methodological Developments (2012–2021) and Remaining Challenges." *International Journal of Biometeorology*, 2022.

J. Salagnac. "Lessons from the 2003 Heat Wave: A French Perspective," 2007.

K. Vanderplanken, J. V. Loenhout, and P. Hazel. "The Health Impact of Heat Waves in Europe: Insights from National Plans and Key Informant Interviews." *European Journal of Public Health*, 2019.

K. Vanderplanken, P. van den Hazel, Michael Marx, A. Shams, D. Guha-Sapir, and J. V. van Loenhout. "Governing Heatwaves in Europe: Comparing Health Policy and Practices to Better Understand Roles, Responsibilities and Collaboration." *Health Research Policy and Systems*, 2021.

Kathleen E. McLean, Melissa Macdonald, and S. Henderson. "Establishing Appropriate Hot Weather Alerting Criteria for British Columbia, Canada." *ISEE Conference Abstracts*, 2018.

Kathleen E. McLean, R. Stranberg, Melissa Macdonald, Gregory R. A. Richardson, T. Kosatsky, and S. Henderson.

“Establishing Heat Alert Thresholds for the Varied Climatic Regions of British Columbia, Canada.” *International Journal of Environmental Research and Public Health*, 2018.

Katya Brooks, O. Landeg, S. Kovats, M. Sewell, and E. OConnell. “Heatwaves, Hospitals, and Health System Resilience in England: A Qualitative Assessment (Summer 2019).” *European Journal of Public Health*, 2024.

Kristie Signer, Summer Formosa, and Tanya Seal-Jones. “Building Community Resilience: The City of Victoria's Approach to Climate Change Adaptation and Extreme Heat Response.” *Journal of Business Continuity and Emergency Planning*, 2023.

M. Boeckmann, G. S. Martinez, and Kendrovski. “Organizing the Institutional Public Health Response to Heat Under Climate Change: A Scoping Review.” *European Journal of Public Health*, 2019.

M. D. Sario, M. Leone, Adele Lallo, M. Davoli, and P. Michelozzi. “Preparedness and Response Plans to Reduce the Health Impact of Heat Waves in Italy,” 2011.

M. Pascal, K. Laaidi, M. Ledrans, E. Baffert, C. Caserio-Schönemann, A. Tertre, Jacques Manach, S. Médina, J. Rudant, and P. Empereur-Bissonnet. “France’s Heat Health Watch Warning System.” *International Journal of Biometeorology*, 2006.

M. Pascal, R. Lagarrigue, Anouk Tabai, Isabelle Bonmarin, Sacha Camail, K. Laaidi, Alain Le Tertre, and Sébastien Denys. “Evolving Heat Waves Characteristics Challenge Heat Warning Systems and Prevention Plans.” *International Journal of Biometeorology*, 2021.

M. Scortichini, F. Donato, M. D. Sario, A. D. Martino, M. Davoli, and P. Michelozzi. “OP II – 6 Temporal Variation in the Effect of Heat and the Role of the Italian Heat Prevention Plan.” *Occupational and Environmental Medicine*, 2018.

M-I Bittner, Eva Franziska Matthies, D. Dalbokova, and B. Menne. “Are European Countries Prepared for the Next Big Heat-Wave?” *European Journal of Public Health*, 2014.

M-I Bittner, F. Matthies, D. Dalbokova, and B. Menne. “Is Europe Prepared for the Next Big Heatwave,” 2013.

Michihiko Tonouchi. “Heat Disorder Information System for Japan in 2012,” 2013.

P. Berry, Anna Yusa, Toni Morris-Oswald, and A. Rogaeva. “Heat Alert and Response Systems in Urban and Rural Communities in Canada,” 2014.

P. Michelozzi, F. D. de’ Donato, A. Bargagli, D. D'ippoliti, M. De Sario, C. Marino, P. Schifano, et al. “Surveillance of Summer Mortality and Preparedness to Reduce the Health Impact of Heat Waves in Italy.” *International Journal of Environmental Research and Public Health*, 2010.

PD Dr. U. Heudorf, and M. Schade. “Heat Waves and Mortality in Frankfurt Am Main, Germany, 2003–2013.”

Zeitschrift Für Gerontologie Und Geriatrie (Print), 2014.

R. Vachat, S. Planton, and M. Gillet. “ADAPTATION TO HEAT WAVES OCCURRENCE IN FRANCE 1,” 2004.

Sara Mehryar, Candice Howarth, and Declan Conway. “Heat Risk Interdependencies in the UK: Implications for Adaptation.” *Earth's Future*, 2025.

Siyu Yu, Kin Long Lei, Dongying Li, You Joung Kim, Mio Nemoto, Sarah Gatson, Makoto Yokohari, and Robert Brown. “Plan Integration for Urban Extreme Heat: Evaluating the Impacts of Plans at Multiple Scales in Tokyo, Japan.” *Urban Climate*, 2024.

Yong-jin Hong, You-ki Min, Sangduk Lee, and Sungmin Choi. “Expanded Orientation of Urban Public Health Policy in the Climate Change Era: Response to and Prevention of Heat Wave in Paris and Seoul: A Brief Review.” *Iranian Journal of Public Health*, 2022.
